# Kinetic of bone turnover markers after osteoporotic vertebral compression fractures in postmenopausal female

**DOI:** 10.1186/s13018-018-1025-5

**Published:** 2018-12-07

**Authors:** Changyu Pan, Xiaoyang Liu, Tao Li, Guodong Wang, Jianmin Sun

**Affiliations:** Department of Spine, Shandong Provincial hospital affiliated to Shandong University, No.9677 Jingshi road, Jinan, 250013 People’s Republic of China

**Keywords:** Bone turnover markers, Osteoporotic vertebral compression fractures, Fracture healing, Postmenopausal women

## Abstract

**Background:**

Osteoporotic fracture occurs mostly at the spine, in which the commonest one is vertebral compression fracture. Bone turnover markers (BTMs) can be applied to assess bone formation and resorption activity. Nevertheless, there are few reports on BTMs changes after osteoporotic vertebral compression fracture. The aim of this study is to investigate the kinetics of bone turnover markers after osteoporotic vertebral compression fractures in postmenopausal female.

**Methods:**

Three hundred nine postmenopausal female patients with osteoporotic vertebral compression fractures were included in the study. Fasting blood samples were obtained to analyze the serum concentration of bone turnover markers including osteocalcin (OC), β-isomerized type I collagen amino-terminal peptide (β-CTX), alkaline phosphatase (ALP), type I procollagen amino-terminal peptides (PINP), calcium, and phosphorus. According to periods long after vertebral fracture, all the cases were divided into seven phases: phase 1 (within 3 days), phase 2 (3 days to 1 week), phase 3 (1 to 2 weeks), phase 4 (2 to 4 weeks), phase 5 (4 to 12 weeks), phase 6 (12 to 24 weeks), and phase 7 (24 weeks to 1 year). Comparisons among the phases and kinetics during the phases were conducted.

**Results:**

All the kinds of BTM’s serum concentration began to increase within 3 days after vertebral fracture in phase 1. Osteocalcin and β-CTX had two peaks, the first one in phase 2 (21.4 ± 6.0 ng/ml and 0.72 ± 0.17 ng/ml, respectively) and the second in phase 6 (25.8 ± 7.5 ng/ml and 0.89 ± 0.23 ng/ml, respectively). The peak of ALP arrived in phase 4 at the value of 123.9 ± 25.7 U/L. PINP reached its peak value (69.50 ± 16.82 ng/ml) in phase 6. Serum phosphorus arrived at its first peak (1.21 ± 0.13 mmol/L) in phase 2 and the second peak (1.23 ± 0.13 mmol/L) in phase 4. Serum calcium reached the first peak (2.30 ± 0.07 mmol/L) in phase 3 and the second peak (2.34 ± 0.08 mmol/L) in phase 5.

**Conclusion:**

The time-dependent variations of BTMs based on the fracture healing process of inflammation, regeneration, and remodeling occur after vertebral fracture. Kinetics of BTMs after vertebral fracture as well as the reference value at each period were established in the present study. It is helpful to assess vertebral fracture healing process according to the kinetics of BTMs.

## Background

Along with the aging population increases to be double in the next decades [1], the incidence of osteoporosis will rise obviously, which makes the bone fragile and liable to fracture [2]. Osteoporotic fracture has become a global health care issue, due to high mortality and costs [3].

Fracture healing can be divided into three phases: the inflammation phase, the regeneration phase, and the remodeling phase. However, osteoporosis could delay fracture healing and impair healing outcomes [4]. Bone biochemical markers (BTMs) help to monitor the bone metabolic rate quantitatively [5–10]. BTMs could enhance the evaluation efficiency for the bone fracture healing, as well as predict early the risk of developing impaired fracture outcomes [9].

BTMs comprise of two categories: bone resorption markers and formation markers. Bone resorption markers are mainly the breakdown products of type I collagen, among which the widely used one is type I collagen amino- or carboxyl-terminal peptides (NTX or CTX). The commonest bone formation marker is type I procollagen amino- or carboxyl-terminal peptides (PICP or PINP) which is the breakdown product dissociated from type I procollagen. The other BTMs include osteocalcin (OC) and alkaline phosphatase (ALP) [10]. Among them, PINP and CTX have been recommended as the main reference markers of bone metabolism in predicting fracture risk and evaluating anti-osteoporosis treatment by the International Osteoporosis Foundation (IOF) and the International Federation of Clinical Chemistry (IFCC) Bone Marker Standards Working Group [11].

Osteoporotic fracture occurs mostly at the spine, in which the commonest one is vertebral compression fracture [12, 13]. Nevertheless, there are few reports on BTMs changes after osteoporotic vertebral compression fracture (OVCF). In this study, we aim to investigate the kinetics of BTMs following OVCF and establish evaluation criteria of the OVCF healing process, through reviewing BTMs values collected at different periods after OVCF.

## Materials and methods

Between January 2015 and June 2018, 309 consecutive postmenopausal female patients for back pain admitted in the authors’ hospital and diagnosed with OVCF were included in the study. All patients went through vertebral fragility fractures, resulting from a simple fall from a standing height or less. Permission to conduct this retrospective study was obtained from the hospital ethics committee. Written informed consent was obtained from each subject involved in the study. The exclusion criteria were (1) secondary or idiopathic osteoporosis, (2) multiple vertebral fractures, (3) liver, intestinal, and renal function dysfunction, (4) undertaking medications known to affect bone metabolism, and (5) nonunion after 24 weeks.

Demographic information was collected including age and period long after vertebral fracture. Fasting blood samples were obtained to analyze the BTMs when admitted to hospital before surgeries and administration of anti-osteoporosis drugs. Samples were stored at − 20 °C before the BTMs analysis done. The serum concentration of total PINP, β-isomerized C-terminal telopeptides (β-CTX), and N-MID osteocalcin (OC_N-Mid_) were all measured by the electrochemiluminescence immunoassay (Elecsys 2010, Roche Diagnostics, Mannheim, Germany), with intra- and interassay coefficients of variation (CVs) of 3.0% and 4.1%, 3.5% and 8.4%, 1.8% and 3.3%, respectively. Serum calcium was measured by photometric color test, serum phosphorus by photometric UV test, and ALP by kinetic color test (Beckman Coulter, Brea, CA, USA). The corresponding intra- and interassay CVs were 0.65% and 0.96%, 1.03% and 1.55%, 1.94% and 4.88%. Bone mineral density (BMD) was measured at the lumbar spine by dual-energy X-ray absorptiometry (DEXA, GE Medical Systems Lunar). According to the period long after vertebral fracture, all of the 309 cases were divided into seven phases: phase 1 (within 3 days), phase 2 (3 days to 1 week), phase 3 (1 week to 2 weeks), phase 4 (2 to 4 weeks), phase 5 (4 to 12 weeks), phase 6 (12 to 24 weeks), phase 7 (24 weeks to 1 year).

The data were processed with SPSS 17.0 statistics software (SPSS Inc., Chicago, IL). Intergroup comparison was analyzed by one-way analysis of variance (ANOVA) tests. *P* value less than 0.05 was considered statistically significant.

## Results

A total of 309 patients were included in the retrospective study. The mean age was 69.0 ± 8.2 years (range 52–90 years). There was no significant difference in age and BMD among the seven phases, which indicated the homogeneous distribution of samples and avoided the effects of age and BMD in normal kinetic of BTMs after OVCFs.

**Fig. 1:**
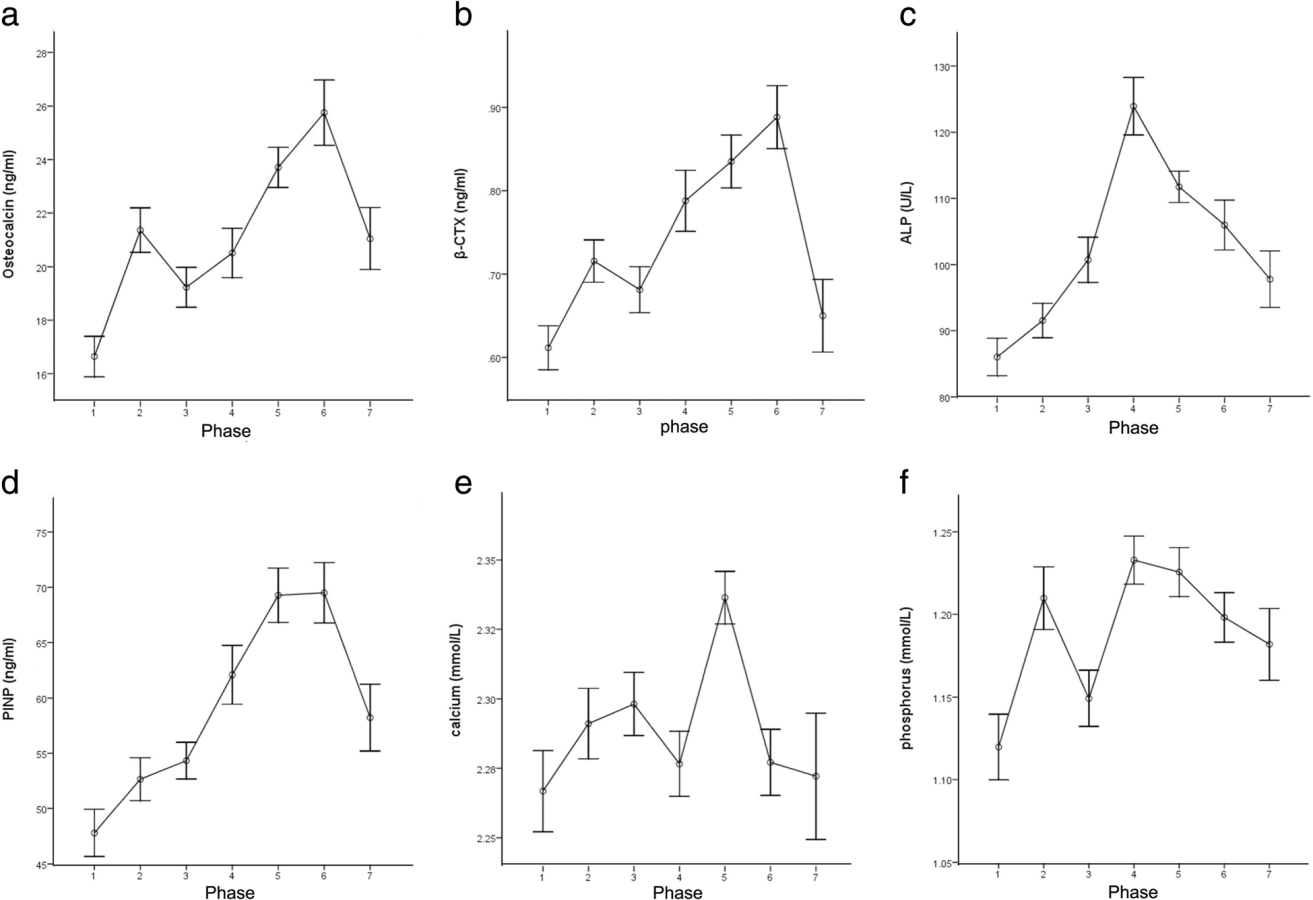
**a**–**f** Kinetics of bone biochemical markers during different time periods after osteoporotic vertebral compression fractures. Results of osteocalcin (**a**), β-isomerized C-terminal telopeptides (β-CTX, **b**), alkaline phosphatase (ALP, **c**), type I procollagen amino-terminal peptides (PINP, **d**), serum calcium (**e**), and serum phosphorus (**f**) are indicated as mean ± standard error of mean (SEM)

All the kinds of BTM’s serum concentration began to increase within 3 days after vertebral fracture (phase 1). However, the existing time and extent of the peak value varied for each kind of BTMs. The changes of BTMs during different periods after vertebral fracture were shown in Fig. 1a–f. Differences of BTMs among seven phases showed statistical significance. Detailed descriptions and comparisons of BTMs among different periods of time after fracture were shown in Table 1. All data conformed to the law of normal distribution; hence, the values were expressed as mean ± standard deviation (SD).

**Table 1:** Comparisons of bone turnover markers and others at different periods of time after OVCFs

Variables	Group 1	Group 2	Group 3	Group 4	Group 5	Group 6	Group 7	*P*
Number	40	46	37	35	75	38	38	
Age (years)	67.1 ± 8.8	71.0 ± 8.9	70.7 ± 9.3	68.1 ± 7.8	68.5 ± 7.4	69.9 ± 7.4	67.6 ± 7.6	0.204
BMD (g/cm^2^)	0.74 ± 0.19	0.69 ± 0.17	0.78 ± 0.14	0.78 ± 0.12	0.70 ± 0.15	0.70 ± 0.20	0.77 ± 0.11	0.138
Osteocalcin (ng/ml)	16.6 ± 4.8	21.4 ± 5.6	19.2 ± 4.6	20.5 ± 5.5	23.7 ± 6.5	25.8 ± 7.5	21.1 ± 7.1	< 0.001
β-CTX (ng/ml)	0.61 ± 0.17	0.72 ± 0.17	0.68 ± 0.17	0.79 ± 0.22	0.84 ± 0.28	0.89 ± 0.23	0.65 ± 0.27	< 0.001
ALP (U/L)	86.0 ± 18.0	91.5 ± 17.8	100.7 ± 20.9	123.9 ± 25.7	111.7 ± 20.5	106.0 ± 23.1	97.8 ± 26.4	< 0.001
PINP (ng/ml)	47.8 ± 13.4	52.6 ± 13.2	54.3 ± 10.1	62.1 ± 15.6	69.3 ± 21.3	69.5 ± 16.8	58.2 ± 18.6	< 0.001
Calcium (mmol/L)	2.27 ± 0.09	2.29 ± 0.09	2.30 ± 0.07	2.28 ± 0.07	2.34 ± 0.08	2.28 ± 0.07	2.27 ± 0.14	0.001
Phosphorus (mmol/L)	1.12 ± 0.13	1.21 ± 0.13	1.15 ± 0.10	1.23 ± 0.09	1.23 ± 0.13	1.20 ± 0.09	1.18 ± 0.13	< 0.001

*OVCFs* osteoporotic vertebral compression fractures, *BMD* bone mineral density, *β-CTX* β-isomerized C-terminal telopeptides, *ALP* alkaline phosphatase, *PINP* type I procollagen amino- terminal peptides

The serum concentration of OC reached the first peak value (21.4 ± 5.6 ng/ml) in phase 2 (3 days to 1 week), then decreased slightly thereafter till 2 weeks after the vertebral fracture, when OC restarted to increase gradually and reached its second peak value (25.8 ± 7.5 ng/ml) phase 6 (12 to 24 weeks). β-CTX had similar kinetics as OC, it reached its first peak (0.72 ± 0.17 ng/ml) in phase 2 and the second peak (0.89 ± 0.23 ng/ml) in phase 6.

ALP began to increase after vertebral fracture gradually and the single peak (123.9 ± 25.7 U/L) arrived in phase 4 (2 to 4 weeks). PINP maintained increasing after 4 weeks since the vertebral fracture; it reached the peak value (69.5 ± 16.8 ng/ml) in phase 6 (12 to 24 weeks). Serum phosphorus concentration reached the first peak (1.21 ± 0.13 mmol/L) in phase 2 (3 days to 1 week) and the second peak (1.23 ± 0.13 mmol/L) in phase 4 (2 to 4 weeks), and then, it declined slightly till 12 weeks after vertebral fracture. Serum calcium reached the first peak value (2.30 ± 0.07 mmol/L) in phase 3 (1 week to 2 weeks). After about 2 week’s decline, a marked increase was observed in phase 5 (4 to 12 weeks), arriving at the second peak value of 2.34 ± 0.08 mmol/L.

## Discussion

Osteoporosis has been becoming a worldwide health issue due to the aging of societies. Osteoporosis is defined as low bone mass and microarchitectural deterioration of bone tissue by the World Health Organization (WHO) [2]. It can increase the bone fragility and the risk of subsequent fracture. For the elderly, the most common type of osteoporotic fracture is OVCF [12, 13]. Most OVCFs belong to fragility fracture, resulting from a simple fall from a standing height or less; even some fractures occur due to coughing, sneezing, or turning over in bed, etc. The early major symptom of OVCF is acute back pain, for which some routine activities could be restricted, such as bending, lifting a heavy object, and sitting from a recumbent position. Generally speaking, to know the clinical manifestations, we can estimate accurately the time when the fracture occurs.

It is well known that there are two categories of osteoporosis: primary and secondary. Most of the patients with primary osteoporosis are postmenopausal female. Bone metabolic processes are uncoupled in postmenopausal female as a character that the elevated bone resorption is much faster than bone formation. It means high bone turnover rate and marked negative calcium balance [14]. In this paper, the objects we investigated were postmenopausal female with primary osteoporosis.

BTMs are good surrogate parameters to monitor the rate of bone formation and resorption. Several studies have investigated the changes of BTMs in serum and urine after lower limb fractures with the aiming to monitor the fracture healing process [5–9]. So far, few data is available in the literature on BTMs’ variation after vertebral fracture, especially for OVCF. Furthermore, most of the previous studies investigated the postoperative changes of BTMs following lower limb fractures. However, operation, especially the instrumentation procedure such as intramedullary nail fixation, affects the bone metabolism markers obviously, because they destruct the bone and then influent BTMs’ normal kinetics [5–8].

The present study is a cross-sectional study designed to avoid the effects of operations and drugs. The authors collected BTMs’ information of OVCF patients before surgeries and administration of anti-osteoporosis drugs. The large scale of samples guarantees the accuracy of the normal kinetics of BTMs after OVCF. Different from the previous studies [5–9], the present study focuses only on OVCF and covers a wide range of period from the onset of the fracture, through the period of fracture repair, to 1 year after the fracture. Meanwhile, the authors selected the subjects before surgeries and administration of anti-osteoporosis drugs and turning out fracture union within 24 weeks, reflecting the normal variances of BTMs in fracture healing process more accurately. Through comparisons among the seven phases, we found that there were no significant differences in age and BMD, which indicated the homogeneous distribution of samples and avoided the effects of age and BMD in the changes of BTMs after OVCFs.

This study selected OC, ALP, and PINP to monitor the bone formation. OC, the most important non-collagen protein produced by the osteoblasts in the bone matrix, is a kind of calcium-binding protein which is dependent on vitamin K and is associated with the mineralization process [15]. ALP is mainly composed of bone and liver isoforms [16], and the presence of bone alkaline phosphatase on the membrane of osteoblastic cells is required for bone mineralization [17]. ALP is capable of monitoring the fracture healing without liver or intestinal disorder [9]. PINP is derived from the degradation of type I procollagen produced by osteoblasts during the conversion of procollagen to collagen [18]. CTX is chosen as bone resorption marker in the present study, which is one kind of breakdown products of type I collagen [10].

Fracture healing comprises of three consecutive phases: inflammation, regeneration, and remodeling phase. But they do not exist one by one; they actually overlap substantially and complete the fracture healing process together [19]. Recently, Scimeca et al. [20] have reported that PTX3 takes an active role in osteoblast proliferation, differentiation, and function; meanwhile, a decreased PTX3 production is observed in osteoporotic patients. According to the effect of PTX3, it is easy to postulate that delayed union or nonunion is related to the low PTXs level which declines the osteogenic capability in vivo.

During the immediate post-fracture period, the bone at fracture surface shows necrosis for 1 or 2 mm due to interruption of blood supply. Phagocytes mop up the necrotic bone and soft tissue, leading to release breakdown products of the bone matrix into the bloodstream, including small degradation fragments of type I collagen, OC, calcium, and phosphorus. Consequently, the level of above assay indices might increase temporarily during the initial stage of bone resorption, as suggested by previous studies [21, 22]. Our results also support the similar changes: the concentrations of serum phosphorus (1.21 ± 0.13 mmol/L), OC (21.4 ± 6.0 ng/ml), and β-CTX (0.72 ± 0.17 ng/ml) reached the first peak values between 3 days and 1 week after vertebral fracture and serum calcium (2.30 ± 0.07 mmol/L) between 1 week and 2 weeks after fracture. It indicated that active osteoclastic removal of the necrotic tissues occurs between 3 days and 2 weeks after the vertebral fracture. It is in accordance with fracture hematoma and inflammation phase demonstrated in biopsies collected within the first 2 weeks after vertebral fractures [23]. Therefore, at the initial stage after fractures, resorption markers reached the first peak concentrations before the increases in bone formation markers, meaning a net loss of bone mass.

In the regeneration phase, the fragment ends are surrounded by cellular tissue to provide a stable structure to benefit skeletal tissue repair. It has been proven that the amount of ALP is accompanied by callus formation [24]. Our results show that ALP reaches maximal concentration (123.9 ± 25.8 U/L) between week 2 and week 4 after fracture. Since the subjects are free from liver or intestinal disorders, ALP value could reflect the bone formation activity [9]. The early soft callus contains mostly fibrous tissue and bone collagen which increases bone tenacity, and the hard callus is growing through continuous mineralization process which enhances bone hardness. According to our results, these bone metabolism markers associated with mineralization increase in turn to benefit calcium and phosphate deposit into the bone matrix possibly. Serum phosphorus (1.23 ± 0.13 mmol/L) reached the second peak between week 2 and week 4, serum calcium (2.34 ± 0.08 mmol/L) between week 4 and week 12, and OC (25.8 ± 7.5 ng/ml) between week 12 and week 24 following vertebral fractures. Histomorphometric analysis of biopsy tissue has demonstrated that active bone matrix synthesis and endochondral bone formation occur at 2–4 weeks and new woven bone formation at 4–6 weeks post-vertebral fracture [23], which achieves consensus with our result on the whole.

With regard to the remodeling phase, which is the final process in fracture healing, it is characterized by a high rate of the bone turnover process. With a series of coupled cycles of bone depositions and resorptions by osteoblasts and osteoclasts, the immature woven bone is replaced by stronger lamellar bone containing regular type I collagen progressively, which accounts for more than 90% of the organic bone matrix [5]. Hence, the levels of markers associated with metabolism of type I collagen will increase correspondingly. Over a period of months or even years, this crude “weld” is remodeled reconstituting normal bone shape [25, 26]. According to our data, PINP (69.50 ± 16.82 ng/ml) and β-CTX (0.89 ± 0.23 ng/ml) reach the maximal concentrations between week 12 and week 24 post-fracture, which is interpreted as the accelerated bone remodeling in this period. Meanwhile, this result also demonstrated that type I collagen played an important role in the remodeling phase. Similar results are reported by Stoffel et al. [5] and Veitch et al. [7], which demonstrate that the PINP and β-CTX rise to a maximum level at week 12 and remain elevated until week 24 secondary to ongoing remodeling stage after tibial fractures. The level of PTX3 should be high in the active bone formation process [20]. However, if PTX3 produced by osteoblasts is insufficient, the remodeling process would be affected, leading to delayed union.

The kinetic of BTMS after OVCFs is helpful to choose the best treatment to promote fracture healing in every phase after OVCF. Conservative or invasive managements are administrated to reduce the acute pain after vertebral fracture. Common conservative treatments include bed rest, painkiller medication, physiotherapy, and bracing [27]. For symptomatic OVCFs without neurological impairment, vertebroplasty and kyphoplasty are effective to relieve the symptoms. They are usually performed as percutaneous minimally invasive vertebral augmentation procedures.

Meanwhile, anti-osteoporotic drugs containing antiresorptive and anabolic agents are essential to facilitate fracture healing. The treatments for osteoporosis should be personalized, because patients could show a great difference in the response to anti-osteoporotic therapy. According to the recent study reported by Valeria et al. [28], C/T-FokI single nucleotide polymorphism (SNP) rather than A/G-BsmI SNP of the vitamin D receptor (VDR) gene could influence the antiresorptive treatment response in postmenopausal osteoporosis.

General variation trends of BTMs after OVCF as well as the reference value at each period are established in our study. According to these criteria of BTMs, we can estimate the rates of bone formation and resorption process and predict the fracture healing outcomes. It is helpful for adapting therapeutic interventions to facilitate fracture healing. However, some deficiencies have to be mentioned. One of the major limitations is the inclusion of a relatively small sample; therefore, further research based on a more larger-scale population will be required in order to draw full conclusions. Moreover, the present utilized BTMs are insufficient to evaluate bone formation and bone resorption, more kinds of BTMs should be taken into account to monitor fracture healing, including bone alkaline phosphatase (BALP) [10], type I procollagen amino-terminal peptides (PIIINP) [18], and Pentraxin 3 (PTX3) [20]. In addition, we only consider the effect of time in fracture healing and neglect other factors that may influence the levels of bone metabolic markers. Therefore, in future study, we will continue to reach more accurate results by differentiating gender, age, BMD, basic diseases, and so on.

## Conclusion

The time-dependent variations of BTMs based on the fracture healing process of inflammation, regeneration, and remodeling occur after vertebral fracture. Kinetics of BTMs after osteoporotic vertebral compression fractures as well as the reference value at each period was established in the present study. It is helpful to assess and monitor better vertebral fracture healing process according to the kinetics of BTMs.

## Abbreviations

ALP: Alkaline phosphatase
BMD: Bone mineral density
BTMs: Bone biochemical markers
CTX: Type I collagen carboxyl-terminal peptides
OC: Osteocalcin
OVCF: Osteoporotic vertebral compression fracture
PINP: Type I procollagen carboxyl-terminal peptides
